# Influence of DNA Mispairing and Abasic Sites on Duplex Dynamics: A Temperature-Jump Infrared Spectroscopy Study

**DOI:** 10.1021/acs.jpclett.6c00991

**Published:** 2026-06-10

**Authors:** Neil T. Hunt, Sophie E. T. Kendall-Price, Ryan Phelps, Gregory M. Greetham, Glenn A. Burley

**Affiliations:** † Department of Chemistry and York Biomedical Research Institute, 8748 University of York, Heslington, York, YO10 5DD, U.K.; ‡ STFC Central Laser Facility, 97008 Rutherford Appleton Laboratory, Didcot, OX11 0QX, U.K.; § Department of Pure and Applied Chemistry, 3527 University of Strathclyde, Glasgow, G1 1XL, U.K.

## Abstract

The dynamics of double-stranded DNA (dsDNA) are central to its biological role as a repository of genetic information. However, under physiological conditions DNA is subject to base mispairing and the formation of abasic sites through processes such as depurination. Such site-specific changes to the established Watson–Crick (WC) architecture would be expected to influence duplex dynamics and so affect key processes including protein binding. Here, we apply temperature-jump infrared spectroscopy to interrogate the relative impact of base pair mismatches and abasic sites on the structural dynamics of a 21-base pair (bp) dsDNA oligomer. The inclusion of an abasic site in the center of the strand leads to destabilization that is manifest as <1 μs time scale disruption of the nearby bases that is not present in fully WC-base paired sequences. Comparing this behavior with sequences featuring mismatches of different sizes shows that a single-bp mismatch causes minimal destabilization, whereas a triple base mismatch results in dynamics that closely mimic those resulting from the presence of an abasic site.

## Graphical abstract

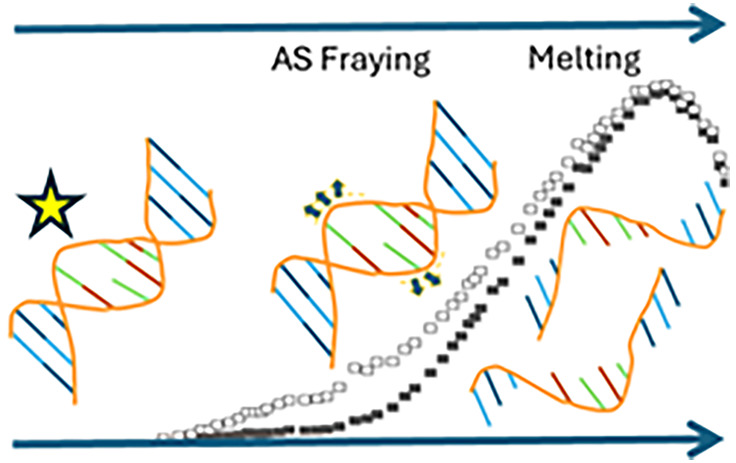

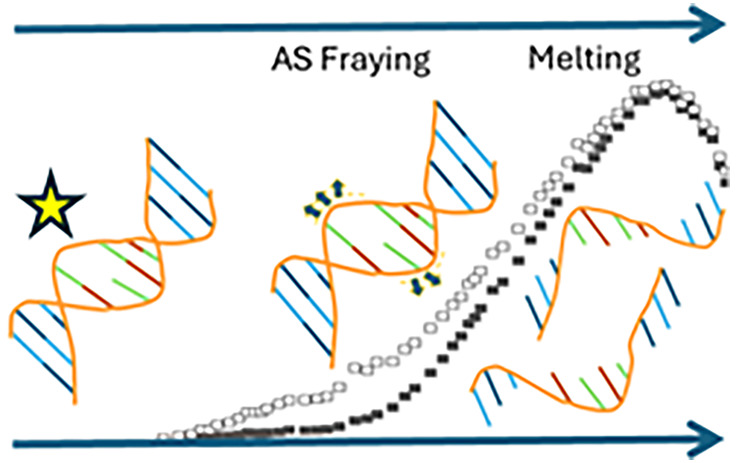

Our traditional view of DNA structure is based on the premise that sequence recognition to form B-type duplexes predominantly follows Watson–Crick (WC) base-pairing rules.^1^ While the predictability of Adenine·Thymine (A·T) and Guanine·Cytosine (G·C) pairings have been used as the foundation for molecular biology for the last 70 years (Figure 1(a)), it has become increasingly apparent that sequences within B-type DNA duplexes can adopt alternative base-pairings and hence tolerate different conformational states under physiological conditions.^2–5^ These alterations in conformational states can arise from noncanonical base-pairing (mismatches, MM)^6–8^ or, more drastically, when a nucleobase is lost as is the case when abasic sites (AS) are formed as a consequence of depurination or DNA repair mechanisms (Figure 1(a)).^9–12^

**Figure 1:**
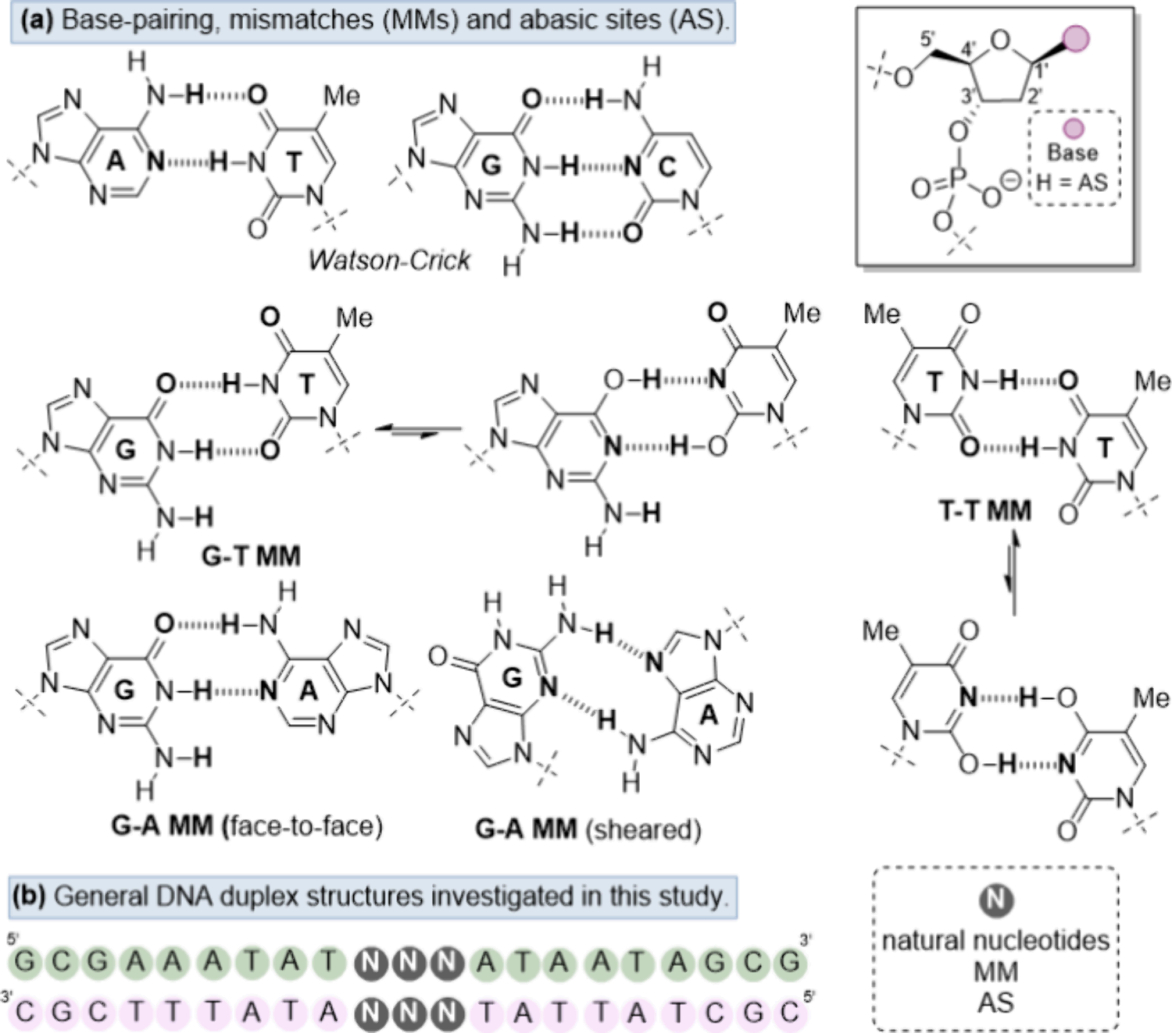
a) Schematic diagrams comparing traditional Watson–Crick base pairing with the mismatch (MM) and abasic site (AS) lesions studied in this work. Note the plasticity of the alternative H-bonding arrangements in the MM pairs. b) Diagram of the sequences studied in this work, identities of the bases labeled N are given in Table 1.

The presence of mismatched base pairs within DNA can result in local conformational perturbations of the B-type duplex structure. In the biological setting these are rapidly reset a few base pairs from the location of the abasic site or mispair, with lifetimes of the conformational changes being on the order of minutes.^13–15^ However, mismatched pairs can evade the DNA repair pathways and, if left unchecked, result in the onset of mutation leading to aging and disease.^16,17^ One prominent example of base mispairing is the G·T mismatch (G·T MM), which induces a two-hydrogen bonded wobble interaction (Figure 1(a)) and can impart local shearing stresses within a duplex.^6,18^ In contrast to the shear stress observed for the larger purine·pyrimidine MM, a T·T MM is far more conformationally flexible, enabling the formation of two types of wobble pairings mediated through a tautomeric intermediate (Figure 1(a)).^19–21^

A more severe alteration of the DNA sequence occurs when an AS is present within a duplex (Figure 1(a)). The reduction in base stacking and hydrogen bond interactions resulting from an AS being present in double-stranded DNA (dsDNA) induces substantial duplex destabilization without significantly altering the overall B-type structure.^22,23^ Consequently, the presence of both MM and AS within DNA duplexes induce differing degrees of local conformational perturbations compared to that observed for B-type duplexes consisting of purely WC base-pairs.

As DNA damage response pathways, as well as recognition, transcription and replication, rely on structural dynamics of dsDNA, including strand separation, it is important to establish an understanding of the impact of how MM and AS influence the WC-architecture. To date, the dynamical interplay between MM and AS within DNA and RNA duplexes has been investigated predominantly using NMR spectroscopy. ^6 19^ ^24,25^ While NMR offers excellent levels of structural insight, the conformational changes of DNA duplexes containing MM or AS are likely to occur over a range of time and length scales, from picosecond-nanosecond base-level fluctuations, to melting and refolding processes taking microseconds-milliseconds, so a technique able to probe these dynamics near MM and AS will add a beneficial new perspective.

Here, we employ temperature (T)-jump infrared (IR) spectroscopy, in which a nanosecond (ns)-duration laser pulse is used to heat the solvent by around 10 °C before a time-delayed IR probe laser pulse monitors changes of the IR spectrum of the DNA in response to the elevated temperature over time scales from ns to milliseconds.^26–30^ The use of IR probing enables signatures from specific base pair types (A·T or G·C) to be differentiated via characteristic spectral markers indicating the disruption of WC base pairing and stacking. In combination with the design of the sequence to be studied, this allows a degree of position-specific insight to be achieved without recourse to labeling or other types of strand modification.

To date, T-jump-IR methods have been applied to investigate the dynamics of DNA melting^27,28–35^ as well as the impact of ligand binding upon the dynamics of the dsDNA double helix,^26,29,36^ including characterization of the allosteric impact of binding at points remote from the ligand site.^26^ Recently, experiments, supported by molecular dynamics simulations, probed the impact of AS inclusion,^9,37^ reporting significant destabilization of 11-bp dsDNA sequences and the occurrence of half-dehybridization, loss of base pairing and stacking on one side of the excised base. ^37^

The motivation for this work is to understand the relative dynamic impacts of noncanonical pairings (MM) and AS lesions. While both are expected to disrupt the overall thermodynamic stability of a duplex, it is not clear how the base stacking and alternative base pairing interactions (Figure 1(a)) in MM will mitigate the resulting perturbation relative to the complete loss of a base (AS). Furthermore, we introduce MM and AS into the center of a 21 base-pair oligodeoxyribonucleotide (ODN, Figure 1(b) and Table 1) to gain an understanding of their impact on longer DNA sequences, more akin to the *in vivo* scenario. We find that introducing an AS into the center of the dsDNA sequence leads to destabilization that is manifest as <1 μs time scale dynamic disruption of WC base pairing. Dynamic perturbation on this time scale is not found in fully base paired sequences. Comparing this dynamic signature to sequences containing MM shows that a single base pair MM (MM1) has minimal impact on the strand dynamics on these time scales, but that a triple base MM (MM3) has a similar destabilizing effect to an AS.

**Table 1:**
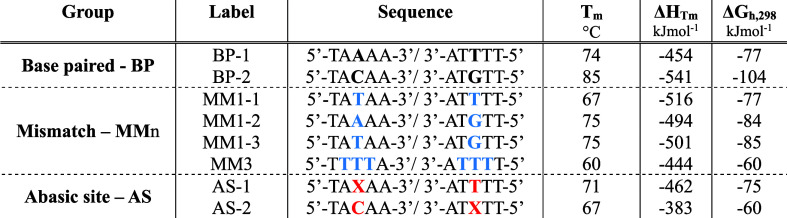
Sequences Studied Alongside Nomenclature and Additional Data^a^

a All sequences are based upon the 5′-GCGAAA­TAT­[**NNN]**AT­AATAGCG-3′ architecture, where the central five bases are listed in the Sequence column of the table. Mismatched bases are highlighted in **blue**; base pairs containing an abasic site (X) are shown in **red**.

The dsDNA sequences studied are shown in Table 1 and Figure 1(b). All are variations on the 21-bp ODN sequence 5′-GCGAAA­TAT­[**NNN]**AT­AATAGCG-3′ hybridized with its complementary sequence. The positioning of G·C pairs at the ends of the duplex and A·T pairs near the middle allows spectroscopic differentiation of dynamics affecting the center and ends of the strand, respectively. This is due to the existence of IR absorption bands arising from vibrational modes of the G and A rings (labeled G_R_ and A_R_) that increase in absorbance upon loss of base pairing and stacking of G·C and A·T base pairs, respectively.^38^ The central bases (NNN) were used to introduce three different single base MM (‘T·G’, ‘T·T’, ‘A·G’, denoted MM1-1 to MM1-3), which mimic the generally isolated nature of MMs within the genome, a triple ‘T·T’ MM (MM3), and two AS (AS-1 and AS-2) in which an A and a G have been excised respectively (Table 1). Two sequences featuring complete WC base pairing (BP-1 and BP-2) were measured to provide direct base paired comparators to the two AS sequences.

All samples were prepared to a dsDNA concentration of 10 mM in 100 mM deuterated phosphate buffer (100 mM NaCl, pD 7). The T-jump measurements were performed using the STFC Central Laser Facility’s ULTRA spectrometer, delivering a 10 °C T-jump to a sample with a 12 μm path length via a method that has been described in detail elsewhere (see also SI).^26–30^

Examples of the results of IR absorption spectroscopy measurements on the sequences in Table 1 as a function of temperature are shown in Figure 2(a–c) and Figure S1. The spectroscopy of dsDNA in the base stretching mode region of the IR spectrum near 1550–1750 cm^–1^ is well-established and the sequences all show bands consistent with predominantly WC-paired structures (Figure S1).^26–35,38^ Examining the region near 1080 cm^–1^_,_ which features absorptions due to stretching modes of the phosphodiester backbone (Figure S2), shows that all sequences exhibit the strong P_2_ symmetric PO_2_^–^ stretching mode at 1085 cm^–1^ that is associated with the double helix structure.^39–41^

**Figure 2:**
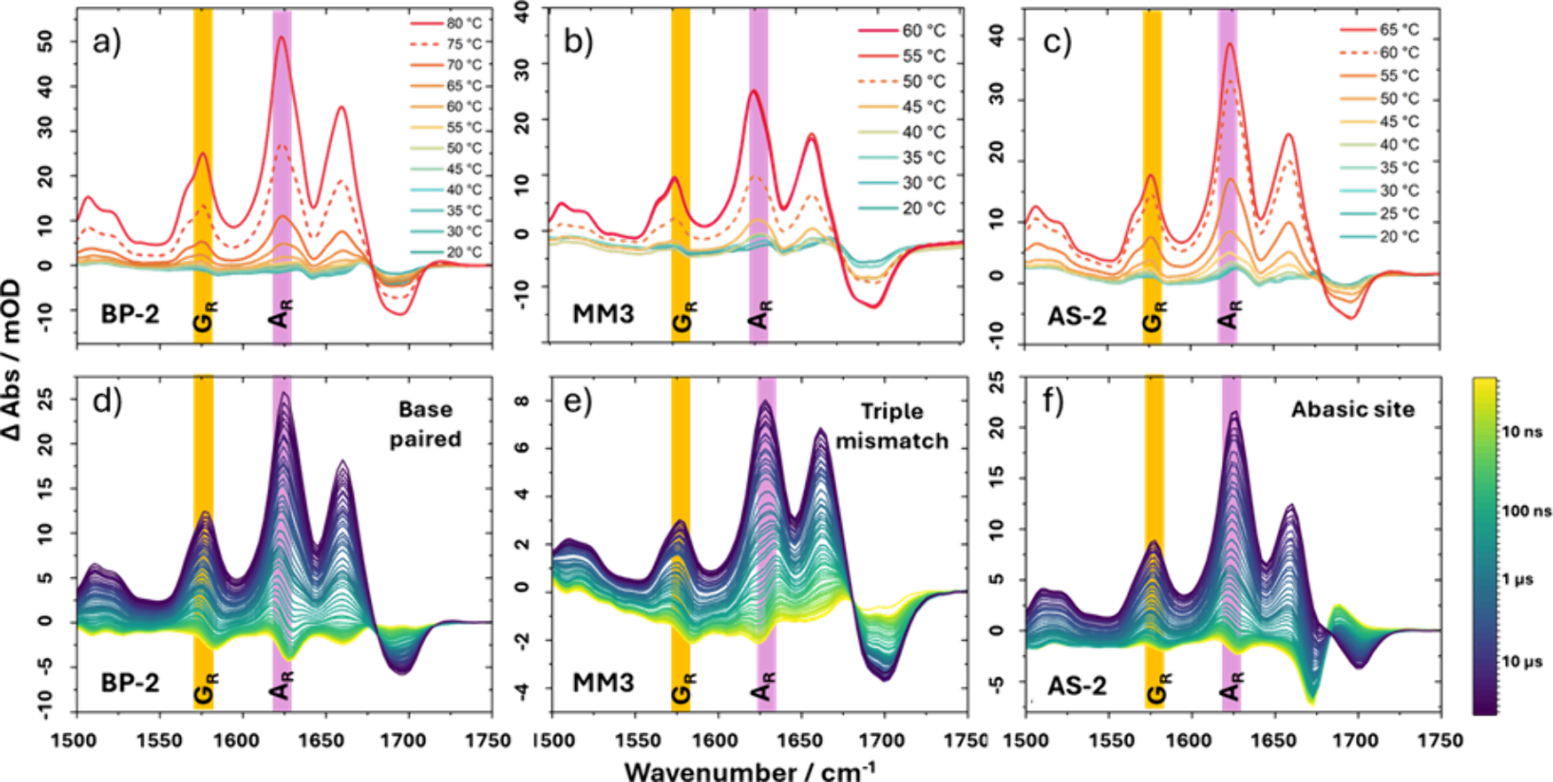
(a–c) IR absorption spectra of dsDNA sequences as a function of temperature. The data are shown as difference spectra equivalent to a T-jump of 10 °C from the temperature indicated. The dashed spectrum was obtained at a temperature equivalent to the data shown in (d–f). (d–f) T-jump IR spectra of the same three sequences shown at a starting temperature of *T*_m_-10 °C for a range of T-jump-probe delay times from 1 ns (yellow) to 40 μs (dark blue), see scale bar. Orange and mauve bars indicate the positions of bands assigned to the G_R_ and A_R_ vibrational modes, which increase in intensity upon disruption of base stacking and pairing for GC and AT base pairs, respectively.

The spectra in Figure 2(a–c) are displayed as difference spectra equivalent to a 10 °C T-jump. Raising the temperature caused an increase in the absorbance of the bands due to the G_R_ (Figure 2(a–c) orange) and A_R_ (mauve) vibrational modes of the G and A bases, respectively. This increase was found to follow a sigmoidal temperature dependence (Figure S1) and is assigned to the loss of base stacking and pairing associated with dsDNA helix melting in accordance with previous reports.^26–35,38^ Fitting to a sigmoidal function allowed extraction of the melting temperature (*T*_m_) of each of the sequences (Figure S1, Table 1). The presence of an extra CG base pair in the center of the sequence (BP-2) induced the highest *T*_m_ value of 85 °C of the sequences studied, as would be expected given the correlation between *T*_m_ and GC content.^42^ With the exception of the sequence featuring the triple MM (MM3_:_ *T*_m_ = 60 °C), little variation in *T*_m_ was observed across the remaining sequences (*T*_m_: 71 ± 4 °C). This is noteworthy given that fully base-paired sequences fall within this range alongside those with both single MM and AS that might have been expected to reduce the thermal stability of the dsDNA.

Direct comparisons of base paired sequences (BP-1/2) and those with corresponding AS reinforce this lack of a general trend in *T*_m_ values. The results for BP-2 and AS-2 show that excision of a G base reduces *T*_m_ by 18 °C. Conversely, BP-1 and AS-1 show that only a 3 °C change accompanies the removal of an A. This is consistent with previous reports showing that damage to a CG pair generally has a greater impact for a given sequence than damage to an AT pair.^23^ It is also noticeable that the changes in *T*_m_ caused by an AS in this work are smaller than have been reported for shorter sequences.^23,37^ This suggests that the greater length of the oligomers studied here may limit the thermal destabilization of the duplex as a whole. Using a two-state model to calculate the Gibbs free energy change for hybridization at 298 K (Δ*G*_h,298_) for each of the sequences (Table 1)^43^ shows similar trends to those from *T*_m_. Introducing an AS to BP-1, to give sequence AS-1, results in a ΔΔ*G*_h_ value (destabilization) of 2 kJmol^–1^, whereas for the AS-2/BP-2 pair, disruption of a GC pair leads to a ΔΔG_h_ value of 44 kJmol^–1^.

In the case of the MM1 sequences, *T*_m_ was found to be relatively insensitive to the presence of a single base pair MM. The largest changes occurred when the CG pair of BP-2 was disrupted by replacing the C with either an A or T (MM1-2, MM1-3), which reduced *T*_m_ by 10 °C (ΔΔ*G*_h_ = 30, 29 kJ mol^–1^). Disruption of the central AT base of BP-1 led to contrasting effects. Replacing the A with a T (MM1–1) caused *T*_m_ to decrease by 7 °C (ΔΔG_h_ = 0 kJ mol^–1^), but replacing T with G (MM1–2) caused a small increase in the *T*_m_ value and an increase in Δ*G*_h_ (ΔΔ*G*_h_ = −7 kJ mol^–1^).

The results of T-jump spectroscopy measurements, in which the temperature of the sample was initially held 10 °C below *T*_m_ of the sequence being studied before a jump of around 10 °C was applied with the T-jump pump laser pulse are shown in Figure 2(d–f) and Figure S3. The spectra are shown as difference spectra relative to the pre-T-jump IR spectrum of the sample and, at late times, are very similar in form to the IR absorption difference spectra (Figure 2(a–c)). Whereas the IR absorption spectra show the equilibrium response of the DNA to increased temperature, the T-jump spectra reveal changes as a function of time and reflect the temporal response of the DNA sample to the sudden rise in temperature. The similarity in the band profile of the two data sets shows that DNA duplex melting is occurring in the time period following the arrival of the pump pulse. This behavior has been described previously.^27,28,31–35,37^

To understand the impact on the structural dynamics of the duplex DNA caused by MM and AS we will focus on the relative temporal behavior of the G_R_ and A_R_ bands of each sequence, which are highlighted in Figure 2(d–f) by orange and mauve boxes, respectively. The dynamics are shown in more detail in Figure 3 where the amplitudes of the G_R_ and A_R_ bands are plotted as a function of T-jump-IR delay time.

**Figure 3:**
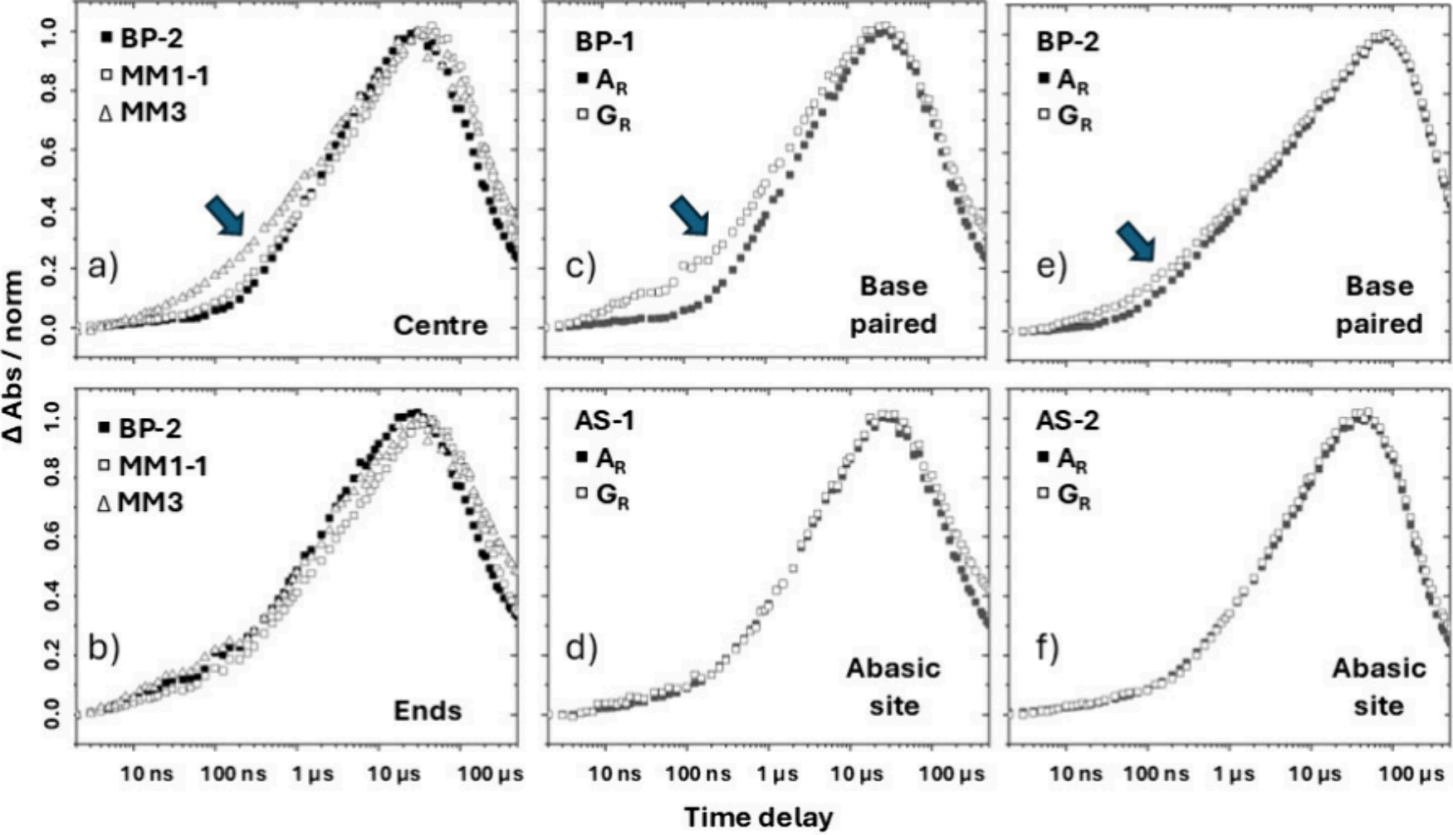
(a, b) Comparison of dynamics observed for the A_R_ (a) and G_R_ (b) bands of representative examples of sequences in the base paired (BP), single mismatch (MM1) and triple mismatch (MM3) groups. The arrow in a) shows the deviation toward faster dynamics observed for the central AT base pairs of the MM3 sequence, whereas b) shows that the ends all behave in a similar manner. (c–f) compare the dynamics observed for the A_R_ and G_R_ bands of BP (c, e) and AS sequences (e, f). The more stable center (A_R_) of the BP sequences relative to the ends (G_R_) is highlighted by the arrows in c) and e), whereas the ends and center behave similarly in d) and f).

For all sequences studied, the G_R_ and A_R_ bands were both observed to rise in intensity following the T-jump, reaching a peak by ∼50 μs. Comparison of the peak change in absorbance of the bands with steady state IR absorption data shows that the strands in the dynamic measurements reach around 80–90% of the expected change in absorbance. This is a result of the time scale for full strand melting being comparable to the cooling time scale of the T-jump in our apparatus. Here, however, rather than the full melting process, we focus upon the dynamics preceding the melting transition, which reflect the behavior of the duplex rather than its separation into single strands.

The rising G_R_ and A_R_ band intensities were found to be well-represented by triple exponential functions between time delays of 1 ns and 20 μs. The latter was chosen as being close to the peak of the rising signal. The results of the fitting process are shown in Figure S4 and Table 2. It should be noted that, although the difference between the starting temperature and *T*_m_ was consistent for all measurements, the absolute value of the starting temperature varied between sequences due to their different *T*_m_ values. Thus, while direct comparisons of dynamic time scales between different parts of a given sequence, for example the GC and AT-rich portions of the sequence, are valid, comparisons between sequences must bear in mind the potential impact of the starting temperature on the measured time scales.

**Table 2:** Results of Fitting T-Jump IR Spectroscopy Data to Triexponential Functions^a^

			time scales			relative contribution		
		sequence	τ_1_, ns	τ_2_, ns	τ_3_, μs	*A*_1_, %	*A*_2_, %	*A*_3_, %
A_R_- center	BP	BP-1	280	1590	8.3	13	26	61
		BP-2	200	1230	11.5	24	28	48
	MM	MM1-1	500	2770	17.9	23	34	42
		MM1-2	443	2070	22.2	13	29	59
		MM1-3	345	1960	14.8	23	27	50
		MM3	31	611	10.7	14	33	52
	AS	AS-1	29	739	8.5	7	36	57
		AS-2	33	727	8.7	5	32	63
		BP-1						
G_R_ - ends	BP	BP-1	10	771	6.7	7	29	65
		BP-2	59	661	9.4	16	34	51
	MM	MM1-1	88	930	9.1	5	37	57
		MM1-2	25	1019	13.0	8	33	59
		MM1-3	42	814	10.6	13	33	55
		MM3	16	925	11.5	15	31	54
	AS	AS-1	17	946	8.8	11	34	55
		AS-2	13	856	8.9	5	33	62

a Uncertainties for τ_1_ are ± 40%, those for τ_2_ are indicated in Figure 4.

Considering the G_R_ mode, which represents the behavior of the bases at the ends of the sequences, the three time scales recovered from the fitting were remarkably consistent across all sequences studied. As the spectral signature, a rise in the intensity of the G_R_ band, is the same for all three time scales there must be a distortion of the stacking and pairing of the bases inherent in all three processes. The first time scale (τ_1_) takes values between 10 and 100 ns and is assigned to thermal fluctuations, such as changes in H-bonding length or solvation, of the DNA bases.^27,28–35^ The third time scale (τ_3_) of around 7–13 μs is assigned to the melting process. It is noted that the time scales for melting observed here are a little shorter than have been reported elsewhere (100 μs for 11 bp oligomers).^37^ We attribute this to the shorter cooling time of our experiment and the restricted time window over which the fitting was performed. This is also consistent with the fact that our samples do not reach a fully melted state prior to cooling. As such, although the time scale for full melting is broadly consistent across our data sets, we view this as poorly defined in numerical terms. The intermediate time scale (τ_2_) has values of 700 ns to 1 μs, an order of magnitude faster than the melting process, which we attribute to distortion preceding melting. Given the location of the GC base pairs in the sequence, we believe that these motions will include end fraying of the sequences. This end fraying time scale is longer than some previous reports, which we attribute to the presence of three GC pairs at the end of the sequence and a much longer overall duplex.^27,28,31,34,35^ However, dynamics of GC base pairs on similar time scales have been observed experimentally and assigned to end fraying, in agreement with MD simulations.^32,33^ It is particularly notable that the behavior of the G_R_ band is insensitive to the nature of the central portion of the sequence. Given that previous studies, using shorter sequences incorporating AS have found destabilization of the full sequence, including the ends, ^37^ this suggests that our oligomers are sufficiently long as to isolate the ends from the center.

In contrast to the G_R_ mode, where all sequences showed similar dynamics, when considering the dynamics of the A_R_ modes we find that the samples fall into two categories (Figure 2, Figure S4 and Table 2). The first group contains the base paired sequences (BP-1&2) and the sequences featuring a single MM (MM1). Here, the τ_1_ and τ_2_ values were considerably longer than observed for the G_R_ modes, with τ_1_ spanning the range 200–500 ns and τ_2_ around 1.5–2.7 μs. The melting time scale, τ_3_, was also found to be slightly longer at 10–20 μs. This suggests a general slowing of the dynamics of the center of these sequences relative to the ends. In the case of the fully base paired sequences (BP) this is perhaps as would be expected. The AT-tracts would be expected to show weaker base pairing interactions than the GC ends on a per base level, but this will be offset by the cooperative benefits of two complete turns of the helix and the associated long sequence of stacked bases. We will consider the implications for the MM1 sequences below.

The second group of sequences comprised the triple MM (MM3) and the two sequences featuring an AS (AS-1&2). For this group of sequences, the τ_1_ time scale was found to be around 30 ns, τ_2_ approximately 700 ns while the melting time scale, τ_3_, was 8.5–10 μs. These values are noticeably similar to the time scales obtained for the G_R_ modes of all of the sequences. The implication is that a fully base paired sequence, or one featuring a single MM, shows a much less dynamic center section of the strand compared to its ends, but the presence of a triple MM or AS destabilizes the central section and leads to faster dynamics, more akin to the ends of the sequence.

The comparative behavior of the two groups of sequences is shown in more detail in Figures 3 and 4. Figure 3(a) and (b) compares the dynamics of a fully base paired sequence (BP-1) with those of MM1-1 and MM3, featuring single and triple T·T MM respectively. Panel (b) shows the marked similarity of the dynamics of the G_R_ modes of the three sequences, which is reflected by the fitting results. Conversely, panel (a) shows that the A_R_ dynamics (strand center) rise more quickly for MM3 than those of the BP and MM1 sequences. This is highlighted by an arrow in panel (a) and reflects the greater thermal disruption caused to the strand center when the (TT)_3_ MM is present (MM3).

**Figure 4:**
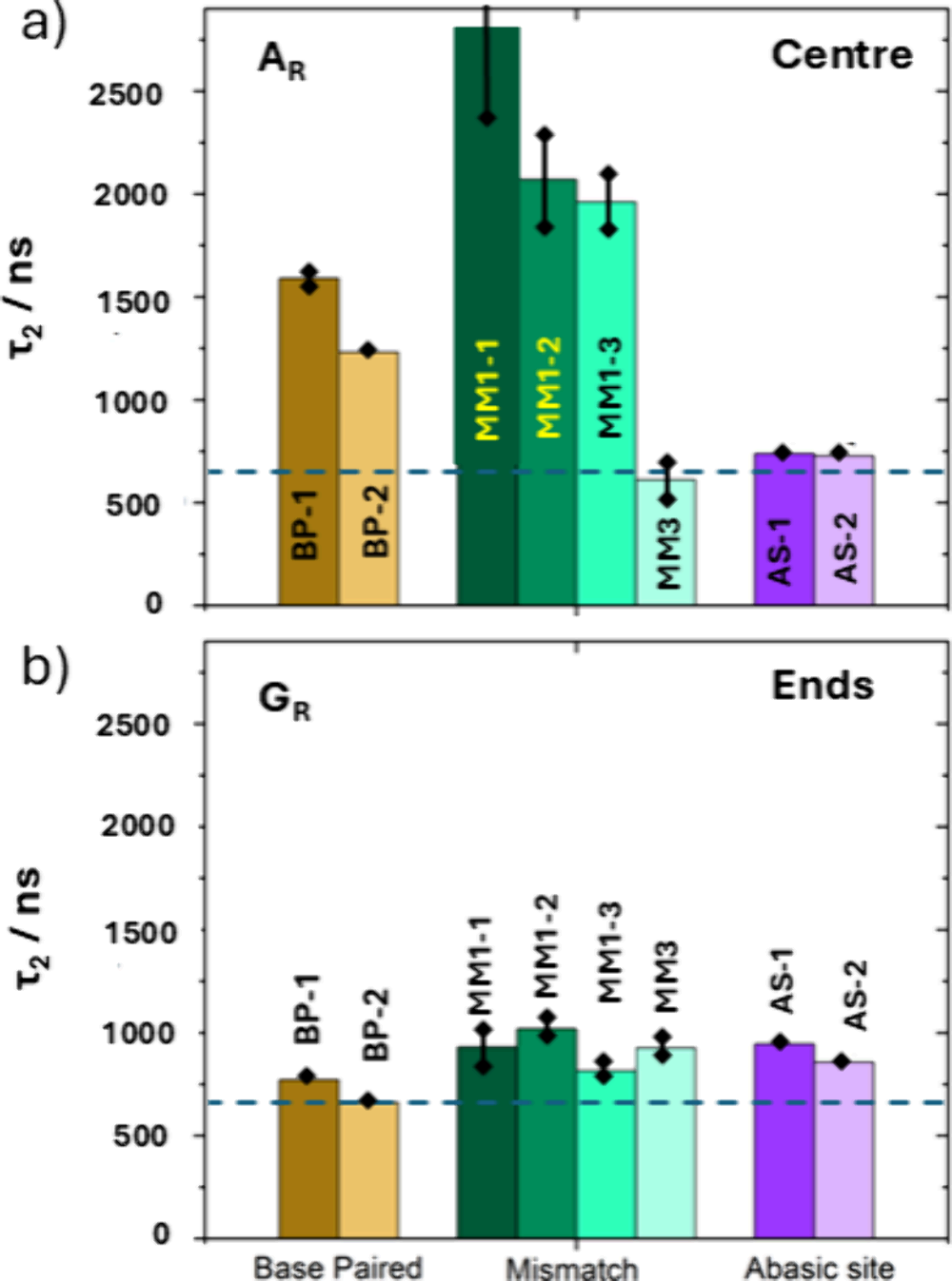
Bar charts comparing the dynamic time scales (τ_2_) obtained from fitting T-jump-IR data (Figures 2 and S4) to triple exponential functions. Panel a) shows that the τ_2_ time scale of the central portion of the dsDNA strand (A_R_) varies strongly with sequence type, being longer in BP and MM1 groups (base paired and single mismatch) but shorter in those with an AS or MM3. By contrast, panel b) shows that the dynamic time scale (τ_2_) observed for the bases at the end of the sequence (G_R_) remains constant across all sequences. The two panels are presented on the same vertical scale showing that the center of the strands (a) are less dynamic (longer time scales) than the ends (b) in BP and MM1 sequences, but similar or more dynamic when a triple mismatch (MM3) or abasic site (AS) is present. The dashed lines are to guide the eye. Error bars are shown in black.

In terms of physically characterizing this distortion, the spectroscopy indicates a rising A_R_ band absorbance, which is consistent with a loss of base stacking and pairing of the AT pairs. While this does not report on the mismatched bases directly, it indicates that the remaining AT pairs become disrupted. It is tempting to describe this as the formation of a bubble in the center of the sequence, however, analysis of the amplitude of the signal at T-jump-IR delay times of ∼1 μs in comparison to the IR absorbance signal under conditions of full melting suggest that the distortion only affects around 1–2 base pairs. Thus, the effect is more consistent with the beginnings of bubble formation, or perhaps, given the similarity of the time scales with the end fraying of the G_R_ mode. This would be consistent with work that has shown that a length of around 20 bp is required for stable bubble formation in the center of a strand.^44^ In addition, studies of DNA breathing and bubble formation dynamics suggest time scales of 10–100 μs, dependent upon bubble size.^45,46^ Taken together, this suggests that we are observing a pseudofraying of the AT base pairs closest to the MM. We stress that the observations of fast A_R_ dynamics for MM3 cannot be explained by disruption of the AT bases near the end of the sequence, for example caused by the (GCG) end fraying process, because these would also be expected to be observed in the fully base paired control sequences, which they are not. In the case of the fully base paired and single MM sequences, the intermediate (τ_2_) dynamics of the A_R_ mode are slowed down by a factor of 2 relative to MM3. While these still represent disruptions of the central base pairs, the time scale is now much closer to the melting process, such that we suggest that these can be viewed as a premelting distortion rather than the faster fraying-type motions that are associated with the larger MM.

Panels (c–f) of Figure 3 directly compare the G_R_ and A_R_ dynamics observed for the two base paired sequences (BP-1&2) with those of the sequences containing an AS based on excising one of the bases in the two BP sequences (AS-1 and AS-2 respectively). In the case of the two AS sequences (Figure 3(d,f)), it is clear that the ends (G_R_) and center (A_R_) show similar dynamics, while for the BP sequences (Figure 3(c,e)) the G_R_ dynamics rise more quickly than those of the centrally located A_R_ modes. This is highlighted by an arrow in panels (c) and (e). We note that the high melting temperature of BP-2 will accelerate the observed dynamics while the central CG base pair will also make the separation of the AT and CG spectral signatures between ends and center less clear-cut, such that the difference is not visually as noticeable in panel (e) as in panel (c).

The results of the dynamic analysis of the T-jump IR data are summarized in Figure 4, in which the τ_2_ values for the center (a) and ends (b) of each sequence are shown on the same scale. A dashed line highlights the same time scale in both panels to guide the eye. The similarity of the G_R_ dynamics across all sequences is clear from Figure 4(b) while the slower dynamics of the A_R_ bands of the BP and MM1 sequences, relative to those of the MM3 and AS sequences is also notable (Figure 4(a)). Perhaps surprising is the fact that the MM1 sequences show a consistently higher τ_2_ value than the BP sequences. The reason for this apparent dynamic stabilization is unclear, though evidence from NMR spectroscopy and molecular dynamics simulations suggest that the distortion of the double helix is limited in the presence of a MM1.^19^ We also note the limited impact upon *T*_m_ and a generally higher ΔG_h_ parameter for the MM1 sequences as compared to BP-1, though in two cases this may arise from the inclusion of an unpaired G base and the situation is reversed relative to BP-2. From our current, data we conclude that the disruption around the MM1 is limited in these sequences, perhaps because of the maintained base stacking in the MM region and the ability to engage flexibly in alternative base pairing conformations (as shown in Figure 1(a)), which resists the tendency of the helix to fray near the MM. Extending the mismatched sequence of bases to three (MM3) overcomes this resistance to deformation and we observe fraying of the AT bases near the MM on a similar time scale to those of the ends of the sequence.

In the case of the two sequences featuring an AS, the destabilizing effect of the excised base, irrespective of its identity or the strand on which it lies leads to a central section of the strand that is at least as dynamic as the ends, with the A_R_ modes showing similar dynamics to the G_R_ equivalent. This is consistent with previous results, which have revealed significant destabilization arising from an AS. ^37^ In contrast to previous studies, however, we see no evidence for a significant degree of dehybridization near the AS on the time scales measured. As with the MM3 sequence, the disruption appears to be limited to a few base pairs, most likely due to similar pseudofraying in the vicinity of the lesion. There are two possible reasons for this observation. The first is that the longer oligomer has provided a greater stabilizing influence over the strand than was possible for the shorter sequences studied previously. It is also possible that the shorter time scale of our instrument leads to different observations, however the time scale for fluctuations is around 2 orders of magnitude shorter than our cooling time scale, making this unlikely. Thus, we conclude that the greater number of helix turns imparts a greater overall stability on the dsDNA, even in the presence of an AS.

In conclusion, the results of T-jump-IR measurements on a range of 21-bp dsDNA sequences featuring single and triple MM and AS show that the sequences fall into two groups according to the relative dynamic behaviors of the ends and the center of the sequence. Fully base paired sequences, and those with a single base MM show fraying of the (GCG) ends on 700 ns time scales while the AT-rich central section shows much slower (1.5–2 μs) premelting dynamics of the duplex. Sequences with a triple MM or AS are significantly destabilized in the center, albeit with no consistent effect on *T*_m_. These show disturbance of the AT bases near the lesion on time scales similar to the (700 ns) fraying time scale of the (GCG) ends. We thus assign these observations to the presence of faster central fraying dynamics of the helix near the MM3 and AS lesions that are not present when the bases are paired or when a single MM is present. The 21-bp length of the sequences effectively isolates the central MM and AS perturbations from the ends of the sequence. We hypothesize that the extended cooperativity of base stacking and, to a lesser extent, alternative base pairing conformations stabilizes sequences featuring a single MM.^47^ Finally, we conclude that the presence of a triple MM engenders a dynamic perturbation to the DNA duplex, manifest as ∼ 1 μs-time scale local dynamics, that is similar to those caused by an AS. We envisage that the differences in the dynamic landscape of DNA duplex conformation could be exploited by nature as a morphological feature to identify the sites of MM and other lesions by DNA damage repair processes.^48,49^

## Supplementary Material



## Notes

Additional experimental details and spectroscopic data (PDF)

## References

[1] Watson J. D., Crick F. H. C. (1953). Molecular structure of nucleic acids - A structure
for deoxyribose nucleic acid. Nature.

[2] Alvey H. S., Gottardo F. L., Nikolova E. N., Al-Hashimi H. M. (2014). Widespread
transient Hoogsteen base pairs in canonical duplex DNA with variable
energetics. Nat. Commun..

[3] Nikolova E. N., Goh G. B., Brooks C. L., Al-Hashimi H. M. (2013). Characterizing
the Protonation State of Cytosine in Transient G·C Hoogsteen
Base Pairs in Duplex DNA. J. Am. Chem. Soc..

[4] Nikolova E.
N., Kim E., Wise A. A., O’Brien P. J., Andricioaei I., Al-Hashimi H. M. (2011). Transient Hoogsteen base pairs in canonical duplex
DNA. Nature.

[5] Leontis N. B., Westhof E. (1998). Conserved geometrical
base-pairing patterns in RNA. Q. Rev. Biophys..

[6] Kimsey I. J., Szymanski E. S., Zahurancik W. J., Shakya A., Xue Y., Chu C. C., Sathyamoorthy B., Suo Z. C., Al-Hashimi H. M. (2018). Dynamic
basis for dG.dT misincorporation via tautomerization and ionization. Nature.

[7] Kimsey I. J., Petzold K., Sathyamoorthy B., Stein Z. W., Al-Hashimi H. M. (2015). Visualizing
transient Watson-Crick-like mispairs in DNA and RNA duplexes. Nature.

[8] Patel D. J., Kozlowski S. A., Ikuta S., Itakura K. (1984). Deoxyadenosine-deoxycytidine
pairing in the d­(C-G-C-G-A-A-T-T-C-A-C-G) duplex - Conformation and
dynamics at and adjacent to the dA.dC mismatch site. Biochemistry.

[9] Ashwood B., Jones M. S., Ferguson A. L., Tokmakoff A. (2023). Disruption
of energetic and dynamic base pairing cooperativity in DNA duplexes
by an abasic site. Proc. Nat. Acad. Sci. U.S.A..

[10] Chen J., Dupradeau F. Y., Case D. A., Turner C. J., Stubbe J. (2008). DNA oligonucleotides
with A, T, G or C opposite an abasic site: structure and dynamics. Nucleic Acids Res..

[11] Lin Z., Hung K. N., Grollman A. P., de los Santos C. (1998). Solution structure
of duplex DNA containing an extrahelical abasic site analog determined
by NMR spectroscopy and molecular dynamics. Nucleic Acids Res..

[12] Wang K. Y., Parker S. A., Goljer I., Bolton P. H. (1997). Solution structure
of a duplex DNA with an abasic site in a dA tract. Biochemistry.

[13] Rossetti G., Dans P. D., Gomez-Pinto I., Ivani I., Gonzalez C., Orozco M. (2015). The structural impact
of DNA mismatches. Nucleic Acids Res..

[14] Imhof P., Zahran M. (2013). The Effect of a G:T
Mispair on the Dynamics of DNA. PLoS One.

[15] Perry J. J. P., Cotner-Gohara E., Ellenberger T., Tainer J. A. (2010). Structural dynamics in DNA damage
signaling and repair. Curr. Opin Struc Bio.

[16] Elez M. (2021). Mismatch Repair:
From Preserving Genome Stability to Enabling Mutation Studies in Real-Time
Single Cells. Cells.

[17] Greenberg M. M. (2014). Abasic
and Oxidized Abasic Site Reactivity in DNA: Enzyme Inhibition, Cross-Linking,
and Nucleosome Catalyzed Reactions. Acc. Chem.
Res..

[18] Hare D., Shapiro L., Patel D. J. (1986). Wobble dG.dT pairing in right-handed
DNA - solution conformation of the d­(C-G-T-G-A-A-T-T-C-G-C-G) duplex
deduced from distance geometry analysis of Nuclear Overhauser Effect
spectra. Biochemistry.

[19] Geng A. N., Roy R., Gu S., Guseva S., Pratihar S., Lee Y., Li L. S., Kimsey I. J., Wilson M. A., Al-Hashimi H. M. (2025). Insight
into the Conformational Ensembles Formed by U-U and T-T Mismatches
in RNA and DNA Duplexes From a Structure-based Survey, NMR, and Molecular
Dynamics Simulations. J. Mol. Bio.

[20] He G. Y., Kwok C. K., Lam S. L. (2011). Preferential
base pairing modes of
T.T mismatches. Febs Letters.

[21] Peyret N., Seneviratne P. A., Allawi H. T., SantaLucia J. (1999). Nearest-neighbor
thermodynamics and NMR of DNA sequences
with internal A·A, C·C, G·G, and T·T mismatches. Biochemistry.

[22] Barsky D., Foloppe N., Ahmadia S., Wilson D. M., MacKerell A. D. (2000). New insights into the structure of
abasic DNA from molecular dynamics simulations. Nucleic Acids Res..

[23] Gelfand C. A., Plum G. E., Grollman A. P., Johnson F., Breslauer K. J. (1998). Thermodynamic
consequences of an abasic lesion in duplex DNA are strongly dependent
on base sequence. Biochemistry.

[24] Nikolova E.
N., Zhou H. Q., Gottardo F. L., Alvey H. S., Kimsey I. J., Al-Hashimi H. M. (2013). A Historical
Account of Hoogsteen Base-Pairs in Duplex
DNA. Biopolymers.

[25] Bothe J. R., Nikolova E. N., Eichhorn C. D., Chugh J., Hansen A. L., Al-Hashimi H. M. (2011). Characterizing
RNA dynamics at atomic resolution using
solution-state NMR spectroscopy. Nat. Methods.

[26] Kendall-Price S. E. T., Nichol R. J. O., Taladriz-Sender A., Phelps R., Malakar P., Greetham G. M., Burley G. A., Hunt N. T. (2025). Long-Range Allosteric
Modulation of DNA Duplex Dynamics Induced by Pyrrole-Imidazole Polyamide
Binding. J. Phys. Chem. Lett..

[27] Howe C. P., Greetham G. M., Procacci B., Parker A. W., Hunt N. T. (2023). Sequence-Dependent
Melting and Refolding Dynamics of RNA UNCG Tetraloops Using Temperature-Jump/Drop
Infrared Spectroscopy. J. Phys. Chem. B.

[28] Howe C. P., Greetham G. M., Procacci B., Parker A. W., Hunt N. T. (2022). Measuring
RNA UNCG Tetraloop Refolding Dynamics Using Temperature-Jump/Drop
Infrared Spectroscopy. J. Phys. Chem. Lett..

[29] Fritzsch R., Greetham G. M., Clark I. P., Minnes L., Towrie M., Parker A. W., Hunt N. T. (2019). Monitoring
Base-Specific Dynamics
during Melting of DNA Ligand Complexes Using Temperature-Jump Time-Resolved
Infrared Spectroscopy. J. Phys. Chem. B.

[30] Greetham G. M., Clark I. P., Young B., Fritsch R., Minnes L., Hunt N. T., Towrie M. (2020). Time-Resolved Temperature-Jump Infrared
Spectroscopy at a High Repetition Rate. Appl.
Spectrosc..

[31] Ashwood B., Tokmakoff A. (2025). Kinetics and dynamics of oligonucleotide
hybridization. Nature Reviews Chemistry.

[32] Ashwood B., Jones M. S., Radakovic A., Khanna S., Lee Y. M., Sachleben J. R., Szostak J. W., Ferguson A. L., Tokmakoff A. (2023). Thermodynamics
and kinetics of DNA and RNA dinucleotide hybridization to gaps and
overhangs. Biophys. J..

[33] Jones M. S., Ashwood B., Tokmakoff A., Ferguson A. L. (2021). Determining Sequence-Dependent
DNA Oligonucleotide Hybridization and Dehybridization Mechanisms Using
Coarse-Grained Molecular Simulation, Markov State Models, and Infrared
Spectroscopy. J. Am. Chem. Soc..

[34] Menssen R. J., Tokmakoff A. (2019). Length-Dependent Melting Kinetics of Short DNA Oligonucleotides
Using Temperature-Jump IR Spectroscopy. J. Phys.
Chem. B.

[35] Sanstead P. J., Tokmakoff A. (2018). Direct Observation of Activated Kinetics
and Downhill
Dynamics in DNA Dehybridization. J. Phys. Chem.
B.

[36] Dale J., Howe C. P., Toncrova H., Fritzsch R., Greetham G. M., Clark I. P., Towrie M., Parker A. W., McLeish T. C., Hunt N. T. (2021). Combining steady state and temperature
jump IR spectroscopy
to investigate the allosteric effects of ligand binding to dsDNA. Phys. Chem. Chem. Phys..

[37] Ashwood B., Jones M. S., Lee Y. M., Sachleben J. R., Ferguson A. L., Tokmakoff A. (2024). Molecular
insight into how the position
of an abasic site modifies DNA duplex stability and dynamics. Biophys. J..

[38] Banyay M., Sarkar M., Graslund A. (2003). A library of IR bands
of nucleic
acids in solution. Biophys. Chem..

[39] Hithell G., Donaldson P. M., Greetham G. M., Towrie M., Parker A. W., Burley G. A., Hunt N. T. (2018). Effect of oligomer length on vibrational
coupling and energy relaxation in double-stranded DNA. Chem. Phys..

[40] Guchhait B., Liu Y., Siebert T., Elsaesser T. (2016). Ultrafast vibrational dynamics of
the DNA backbone at different hydration levels mapped by two-dimensional
infrared spectroscopy. Struct. Dyn..

[41] Siebert T., Guchhait B., Liu Y., Costard R., Elsaesser T. (2015). Anharmonic
Backbone Vibrations in Ultrafast Processes at the DNA-Water Interface. J. Phys. Chem. B.

[42] Breslauer K. J., Frank R., Blocker H., Marky L. A. (1986). Predicting DNA Duplex
Stability from the Base Sequence. Proc. Nat.
Acad. Sci..

[43] Marky L. A., Breslauer K. J. (1987). Calculating thermodynamic data for transitions of any
molecularity from equilibrium melting curves. Biopolymers.

[44] Zeng Y., Montrichok A., Zocchi G. (2004). Bubble nucleation and cooperativity
in DNA melting. J. Mol. Bio.

[45] Phelps C., Lee W., Jose D., von Hippel P. H., Marcus A. H. (2013). Single-molecule
FRET and linear dichroism studies of DNA breathing and helicase binding
at replication fork junctions. Proc. Nat. Acad.
Sci..

[46] Altan-Bonnet G., Libchaber A., Krichevsky O. (2003). Bubble dynamics in double-stranded
DNA. Phys. Rev. Lett..

[47] Yakovchuk P., Protozanova E., Frank-Kamenetskii M.
D. (2006). Base-stacking and base-pairing
contributions into thermal stability of the DNA double helix. Nucleic Acids Res..

[48] Timmins J. (2023). Recognition
of DNA Lesions. International Journal of Molecular
Sciences.

[49] Yang W. (2008). Structure
and mechanism for DNA lesion recognition. Cell
Research.

