# Supplementary material for: Influence of DNA Mispairing and Abasic Sites on Duplex Dynamics: A Temperature-Jump Infrared Spectroscopy Study
Source: J Phys Chem Lett. 2026 Jun 10;17(25):7111–9. doi: 10.1021/acs.jpclett.6c00991 (PMC13312440; doi:10.1021/acs.jpclett.6c00991)
Supplement: Supplementary file 1 [file jz6c00991_si_001.pdf]

Influence of DNA Mispairing and Abasic Sites on Duplex Dynamics: A Temperature-jump Infrared Spectroscopy Study

Neil T. Hunt,[1] Sophie E.T. Kendall-Price,[1] Ryan Phelps,[2] Gregory M. Greetham,[2] Glenn A. Burley,[3]**

1) Department of Chemistry and York Biomedical Research Institute, University of York, Heslington, York, YO10 5DD, UK

2) STFC Central Laser Facility, Rutherford Appleton Laboratory, Didcot, OX11 0QX, UK

3) Department of Pure and Applied Chemistry, University of Strathclyde, Glasgow, G1 1XL, UK

Corresponding Authors

* neil.hunt@york.ac.uk; glenn.burley@strath.ac.uk

INFRARED SPECTROSCOPY

For all spectroscopy measurements, samples were prepared to a concentration of 10 mM in 100 mM deuterated phosphate buffer (100 mM NaCl, pD 7). A 15−20 μL aliquot of the solution was placed in a temperature-controlled cell (Harrick, ±1 °C) equipped with CaF_2 windows and a 12 μm path length.

IR absorption spectra were measured using a Bruker Vertex 70 Fourier transform (FT)-IR spectrometer with a resolution of 1 cm^{-1}. The T-jump measurements were performed using the STFC Central Laser Facility's ULTRA spectrometer, using a method that has been described in detail elsewhere.[1] Briefly, a 4 ns-duration (1 kHz, chopped to 500 Hz) T-jump pump pulse, tuned to the high frequency wing of the OD stretching vibrational band of the solvent was used to deliver a rapid increase in temperature from the initial value (T_0) set by the temperature-controlled sample cell. A time-delayed probe pulse (50 fs) tuned to coincide with the base stretching modes of the dsDNA sample near 1600 cm^{-1} was used to monitor the evolution of the sample following the T-jump. The T-jump pump-probe time delay was continuously variable from ns to 100 μs using a digital pulse delay generator, with the 100 μs to ms delays measured by subsequent probe pulses from the probe laser, which operated at a repetition rate of 10 kHz. Calibration of the T-jump spectrometer with a solution of trifluoroacetic acid in D_2O, using previously reported methods,[1] established that the T-jump obtained was 12 °C.

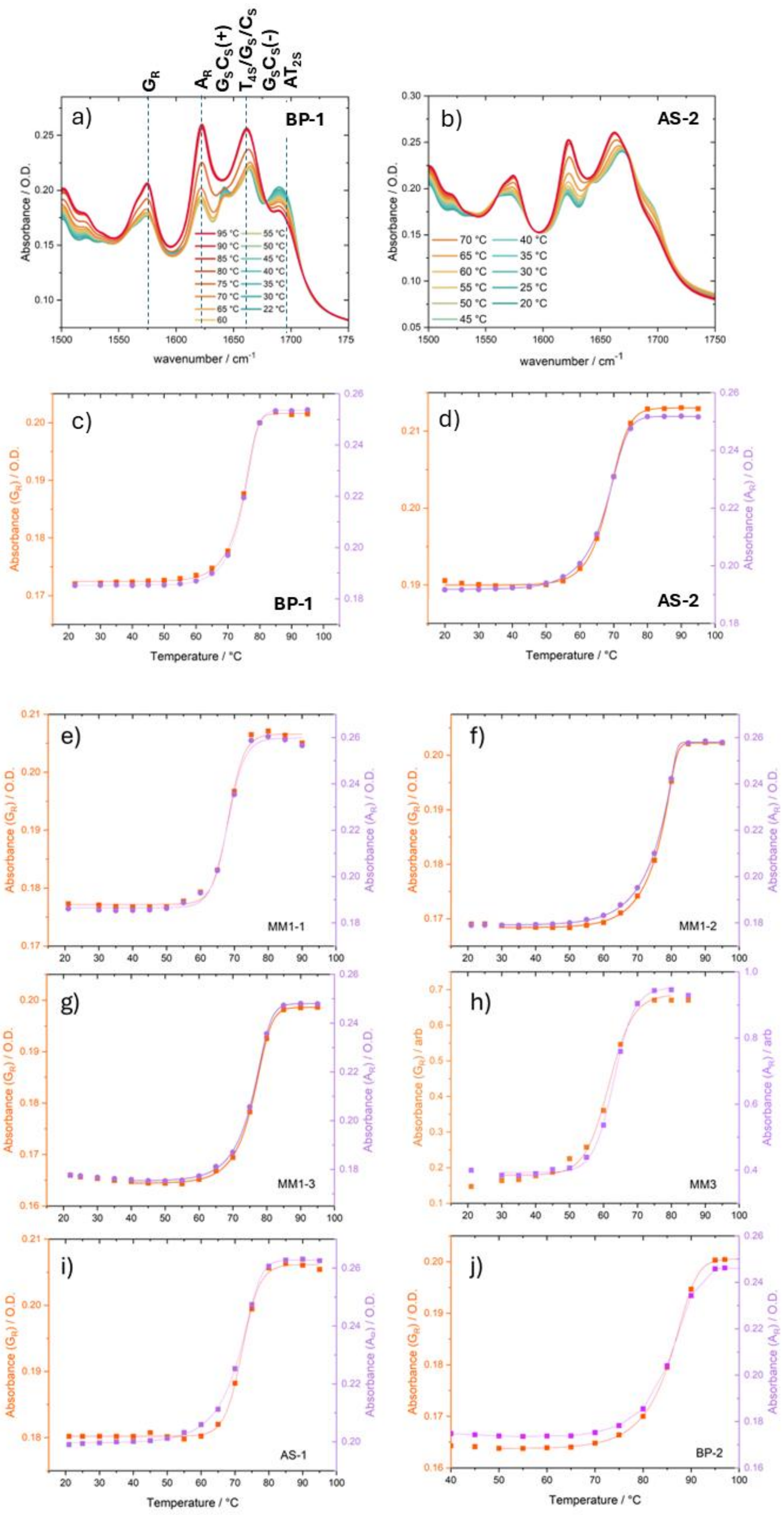

G_R
A_R
G_S C_S(+)
T_4S/G_S/C_S
G_S C_S(-)
AT_2S
a)
BP-1
b)
AS-2
Absorbance / O.D.
wavenumber / cm-1
95 °C
90 °C
85 °C
80 °C
75 °C
70 °C
65 °C
60
55 °C
50 °C
45 °C
40 °C
35 °C
30 °C
22 °C
25 °C
20 °C
c)
d)
e)
f)
g)
h)
i)
j)
Absorbance (G_R) / O.D.
Absorbance (A_R) / O.D.
Absorbance (G_R) / arb
Absorbance (A_R) / arb
Temperature / °C
MM1-1
MM1-2
MM1-3
MM3
AS-1
BP-2

Figure S1 (prev page)**:** (a, b) Representative FTIR spectra shown as a function of temperature for sequences BP-1 and AS-2 (Table 1). (c-j) Show the changes in the intensity of the A_R (mauve) and G_R (orange) bands for all sequences as a function of temperature. Lines show the results of fitting to sigmoidal functions (see text).

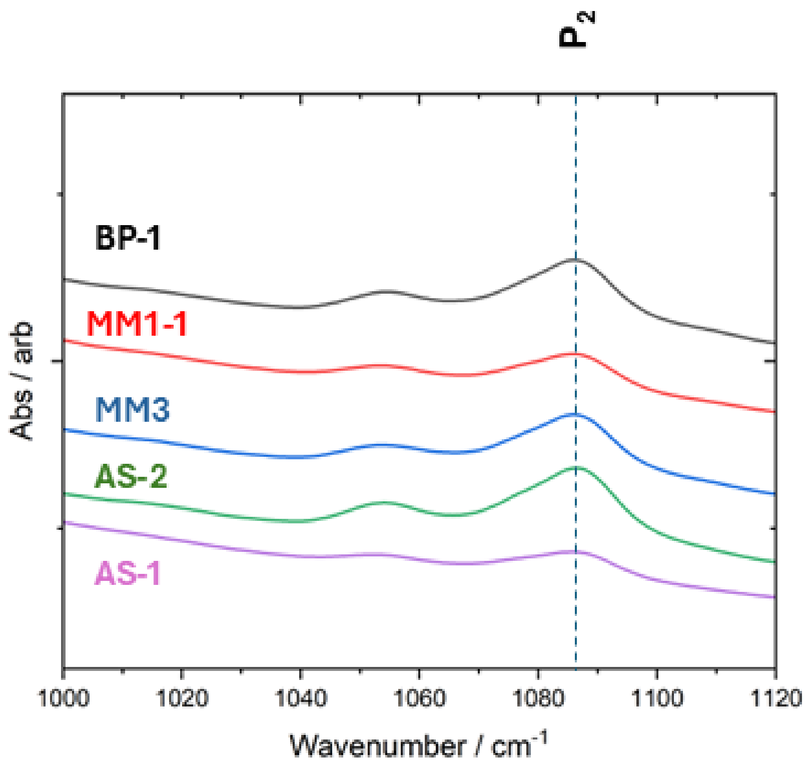

Figure S2: FTIR spectra of a selection of sequences in the phosphate backbone stretching mode region of the spectrum.

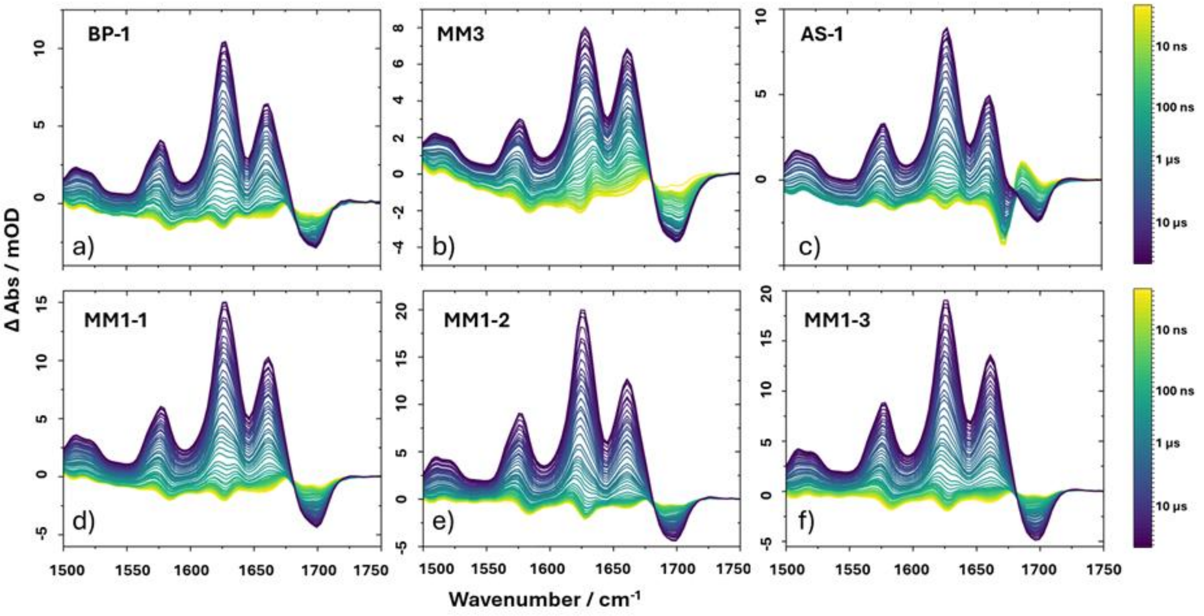

Figure S3: Representative T-jump IR spectra for a selection of sequences (see Table 1). The spectra show T-jump-IR probe delay times ranging from 2 ns (green) to 40 µs (dark blue), see scale bar.

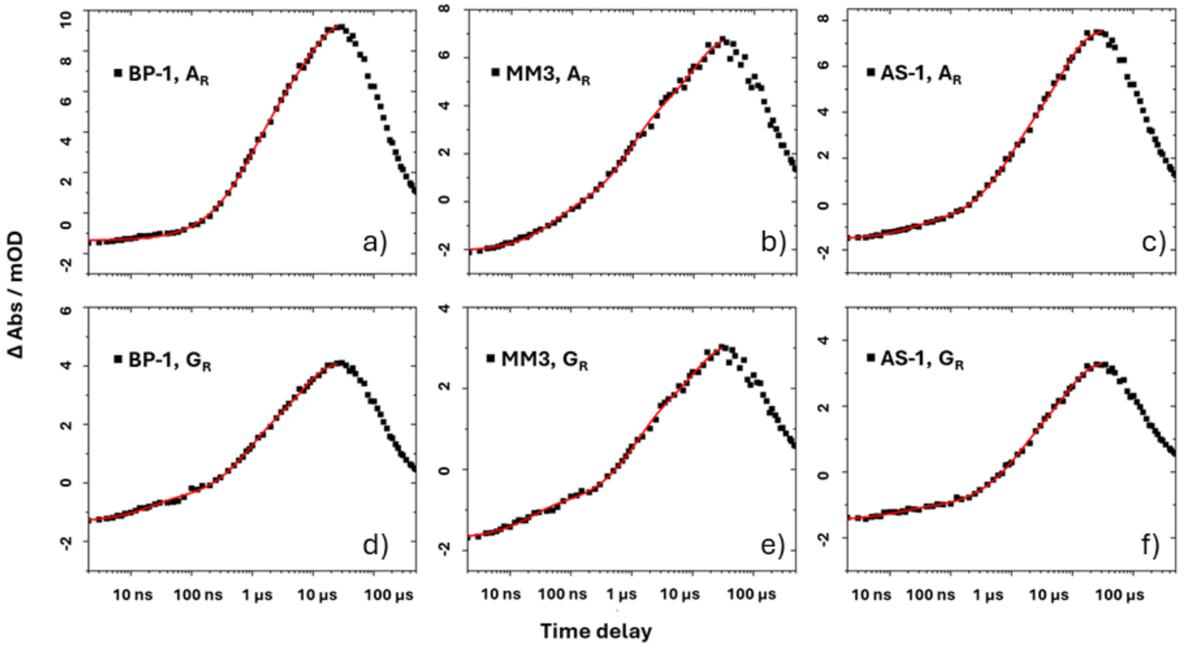

Figure S4: Representative fits to dynamics of A_R (a-c) and G_R (d-f) bands from T-jump IR spectra for a selection of sequences (see Table 1). Points indicate experimental data, red lines show the results of fitting to a tri-exponential function (see text).

REFERENCES

1) Greetham, G. M.; Clark, I. P.; Young, B.; Fritsch, R.; Minnes, L.; Hunt, N. T.; Towrie, M. Time-Resolved Temperature-Jump Infrared Spectroscopy at a High Repetition Rate. *Appl. Spectrosc.* **2020**, *74*, 720–727
